# On the Pentapeptide as the Measurement Unit in Immunology

**DOI:** 10.1055/s-0044-1779041

**Published:** 2024-01-18

**Authors:** Darja Kanduc

**Affiliations:** 1 Department of Biosciences, Biotechnologies, and Biopharmaceutics, University of Bari, Bari, Italy

**Keywords:** epitope dimensions, pentapeptides, vaccines

## Abstract

This communication concerns a crucial query in immunology, that is, the dimension of an epitope. The issue has essential implications in vaccine formulations.

## Introduction

The contact surface between the membrane-bound B cell receptor and an antigenic epitope is made up of five amino acid (aa) residues. It is a pentapeptide.

This notion was defined with mathematical precision in 1939, when Landsteiner and van der Scheer 1 demonstrated by inhibition reactions that pentapeptides serve as specific immune determinants in the generation of specific antibodies. Those studies are a fundamental step in immunology. Indeed, as Landsteiner stated: “ *one cannot safely offer an opinion concerning the specific groups of proteins (‘determinants’) as long as it is not known what the size of such a determinant can be* .” 2

Then, analysis of the binding energy in antigen–antibody complexes, site-directed mutagenesis, and epitope mapping experiments definitively validated the pentapeptide as a minimal antigenic determinant. 3 4 5 6 7

**Fig. 1:**
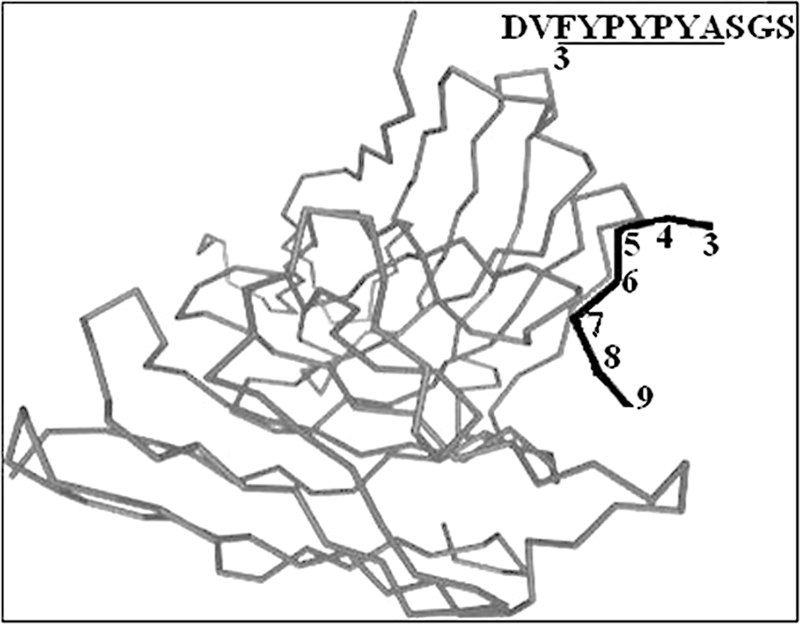
Crystal structure of mimicry-recognizing 2D10 scFv (gray line) with peptide DVFYPYPYASGS (black line). (Source: www.rcsb.org/3d-view/4H0H/1 ; authors: Tapryal S, Gaur V, Kaur KJ, Salunke DM. DOI: 10.2210/pdb4H0H/pdb.)

Moreover, in the early 2000s, Salunke's lab “photographed” the interaction of 2D10 single-chain variable fragment with the dodecapeptide DVFYPYPYASGS ( Fig. 1 , https://doi.org/10.2210/pdb4H0H/pdb ). It can be seen that the interaction involves the five residues numbered 4 to 8 along the black line and corresponding to the sequence YPYPY (see inset in Fig. 1 ).

However, in spite of such incontrovertible data, current immunology reports aa sequences of whatsoever length as epitopes. One example is the sequence AAGKATTEEQKLIEDINVGFKAAVAAAASVPAA, 33 aa long, which is cataloged as an epitope in the Immune Epitope Database ( https://www.iedb.org/ ) with the IEDB ID 160.

## Conclusion

This communication is of relevant importance for defining safe and effective vaccine formulations. Indeed, only pentapeptide-based vaccines can elicit specific antibodies capable of reacting with the full-length antigen without the risk of cross-reactivity and adverse events. 8 9
